# Mitophagy Activation via the YAP/Parkin Pathway Underlies the Neuroprotective Action of Tetramethylpyrazine in Cerebral Ischemia/Reperfusion Injury

**DOI:** 10.3390/biom16030429

**Published:** 2026-03-13

**Authors:** Lanxi Xu, Meiyu Wang, Yan Feng, Sihan Wang, Yihan Qian, Weiru Jiang, Jiadong Xu, Yan Fang, Yani Zhang, Lisheng Chu

**Affiliations:** Department of Physiology, Zhejiang Chinese Medical University, Hangzhou 310053, China; 20211008@zcmu.edu.cn (L.X.); 202311115911081@zcmu.edu.cn (M.W.); 202111115911076@zcmu.edu.cn (Y.F.); 202312210906035@zcmu.edu.cn (S.W.); 202312210906008@zcmu.edu.cn (Y.Q.); 20221104@zcmu.edu.cn (W.J.); 20201007@zcmu.edu.cn (J.X.); 20141040@zcmu.edu.cn (Y.F.)

**Keywords:** tetramethylpyrazine, cerebral ischemia/reperfusion injury, mitophagy, YAP/Parkin, neuroprotection

## Abstract

Background: Mitophagy is a critical mitochondrial quality control mechanism that limits neuronal injury following cerebral ischemia/reperfusion injury (CI/RI). Tetramethylpyrazine (TMP), a bioactive alkaloid from *Ligusticum chuanxiong* Hort., exhibits neuroprotective effects in cerebrovascular disorders. However, whether these effects involve mitophagy regulation remains unclear. Methods: CI/RI was induced using a middle cerebral artery occlusion/reperfusion (MCAO/R) model in mice and an oxygen–glucose deprivation/reoxygenation (OGD/R) model in HT22 cells. Neurological function, infarct volume, mitochondrial function, and mitophagy-related markers were assessed. Pharmacological inhibitors and genetic manipulation of YAP and Parkin were used to investigate underlying mechanisms. Results: TMP treatment significantly reduced infarct volume and improved neurological deficits in MCAO/R mice, accompanied by enhanced mitophagy, as indicated by increased mitochondrial LC3 recruitment and Parkin expression. In OGD/R-injured HT22 cells, TMP promoted mitophagosome and mitolysosome formation, reduced mitochondrial reactive oxygen species, and restored mitochondrial membrane potential. Inhibition of mitophagy with Mdivi-1 attenuated TMP-mediated neuroprotection. Mechanistically, TMP promoted YAP nuclear localization, and inhibition of YAP or silencing of Parkin abolished TMP-induced mitophagy, while Parkin overexpression restored mitophagy under YAP inhibition. Conclusions: TMP alleviates CI/RI by promoting mitophagy through the YAP/Parkin signaling pathway, suggesting mitophagy modulation as a potential therapeutic strategy for ischemic brain injury.

## 1. Introduction

Cerebral ischemia/reperfusion injury (CI/RI) is a major pathological event underlying ischemic stroke and remains a leading cause of mortality and long-term neurological disability worldwide [1,2]. Although timely reperfusion is essential for restoring cerebral blood flow, it paradoxically exacerbates neuronal injury through a cascade of pathological processes, including oxidative stress, mitochondrial dysfunction, and cell death [3,4]. Accumulating evidence indicates that mitochondrial impairment is a central determinant of neuronal vulnerability during CI/RI, highlighting the need to identify effective strategies to preserve mitochondrial homeostasis and neuronal survival [5,6].

Mitophagy, a selective form of autophagy that eliminates damaged mitochondria, plays a critical role in maintaining mitochondrial quality control under stress conditions [7,8]. By removing dysfunctional mitochondria, mitophagy limits excessive mitochondrial reactive oxygen species production, preserves mitochondrial membrane potential, and prevents activation of apoptotic signaling pathways [9]. Dysregulation of mitophagy has been implicated in the pathogenesis of CI/RI, whereas restoration of mitophagic activity has been shown to confer neuroprotection in experimental models [10,11,12]. Among the regulatory mechanisms of mitophagy, the E3 ubiquitin ligase Parkin is a key mediator that orchestrates the recognition and clearance of damaged mitochondria [13]. However, the upstream signaling pathways governing Parkin-dependent mitophagy during CI/RI remain incompletely understood.

Tetramethylpyrazine (TMP), a bioactive alkaloid isolated from *Ligusticum chuanxiong* Hort, has long been used in the treatment of cerebrovascular disorders and has demonstrated antioxidant, anti-inflammatory, and anti-apoptotic properties in experimental stroke models [14,15]. In our previous study, we showed that TMP-treated bone marrow-derived mesenchymal stem cells significantly reduced neuronal apoptosis and promoted vascular remodeling and neurogenesis during the chronic recovery phase following cerebral ischemia in rats [16,17,18]. Nevertheless, whether TMP exerts direct neuroprotective effects by modulating mitophagy during the acute phase of CI/RI, and the underlying molecular mechanisms involved, remain largely unexplored.

Yes-associated protein (YAP) is a transcriptional co-activator of the Hippo signaling pathway and has recently been implicated in mitochondrial regulation and cell survival [19,20,21]. Emerging evidence suggests that YAP activity may influence mitochondrial quality control and autophagy-related processes [22,23], yet its role in mitophagy regulation during CI/RI remains largely unexplored. In particular, whether YAP functions as an upstream regulator of Parkin-mediated mitophagy in the context of ischemic brain injury has not been clarified.

In the present study, we investigated the role of TMP in regulating mitophagy following CI/RI and the involvement of the YAP/Parkin signaling axis. We demonstrate that TMP enhances mitophagy, preserves mitochondrial integrity, and confers neuroprotection. Furthermore, we provide evidence that YAP activity is required for TMP-induced Parkin upregulation and mitophagy activation. Collectively, these findings identify a YAP/Parkin–mitophagy pathway underlying the neuroprotective actions of TMP and suggest mitophagy modulation as a potential therapeutic strategy for ischemic brain injury.

## 2. Materials and Methods

### 2.1. Animals

Adult male C57BL/6 mice (8–10 weeks old, body weight 22–25 g) were obtained from SIPPR/BK Laboratory Animal (Shanghai, China). Mice were housed under specific pathogen-free conditions at 22 ± 2 °C with a 12 h light/dark cycle. Adult male mice were used to minimize potential confounding effects of sex hormones in cerebral ischemia models.

### 2.2. Middle Cerebral Artery Occlusion/Reperfusion (MCAO/R) Model

Transient focal cerebral ischemia was induced using the intraluminal filament method. Mice were anesthetized with isoflurane. A nylon monofilament (Cinontech Co., Ltd., Beijing, China; catalog no. 1620A2) was inserted via the right common carotid artery and advanced through the internal carotid artery to occlude the origin of the middle cerebral artery (approximately 9–11 mm from the carotid bifurcation). After 60 min of occlusion, the filament was withdrawn to allow reperfusion. Sham-operated mice underwent identical surgery without filament insertion.

### 2.3. Animal Grouping and Drug Administration

Mice were randomly assigned to Sham, MCAO/R, MCAO/R + TMP, or MCAO/R + TMP + Verteporfin (VP) groups. TMP (CAS No. 1124-11-4) was administered intraperitoneally at 10, 20, or 40 mg/kg immediately upon reperfusion, as described previously [17,24]. To inhibit YAP activity in vivo, 10 mg/kg VP (MedChemExpress, Monmouth Junction, NJ, USA; catalog no. HY-B0146) was administered intraperitoneally at the onset of reperfusion. Sham and MCAO/R groups received vehicle only. All animals were sacrificed 24 h after reperfusion for subsequent analyses.

### 2.4. Neurological Assessment and Grip Strength Test

Blinded investigators performed all assessments 24 h after MCAO/R. Neurological deficits were evaluated using the Longa score (0–4): 0, no deficit; 1, incomplete extension of the left forepaw; 2, leftward circling; 3, falling to the contralateral side; and 4, reduced consciousness with no spontaneous walking. Forelimb grip strength was measured using a grip strength meter by allowing mice to grasp a metal grid and gently pulling them backward by the tail until release. Grip strength was defined as the mean peak force at release.

### 2.5. TTC Staining and Infarct Volume Analysis

Mice were sacrificed and brains were sectioned into 2 mm coronal slices. Sections were incubated in 2% 2,3,5-Triphenyltetrazolium chloride (TTC; Sigma-Aldrich, St. Louis, MO, USA; catalog no. T8877) at 37 °C for 15 min and fixed with 4% paraformaldehyde overnight. The infarct volume was quantified using ImageJ 1.54 software (NIH, Bethesda, MD, USA) and corrected for edema. Infarct volume (%) = [contralateral hemisphere volume − (ipsilateral hemisphere volume − infarct volume)]/contralateral hemisphere volume × 100%. Edema degree (%) = (ipsilateral hemisphere volume − contralateral hemisphere volume)/contralateral hemisphere volume × 100%.

### 2.6. Cell Culture and Oxygen–Glucose Deprivation/Reoxygenation (OGD/R) Model

HT22 mouse hippocampal neuronal cells were cultured in high-glucose DMEM medium supplemented with 10% FBS, 100 U/mL penicillin, and 100 µg/mL streptomycin. OGD/R was performed by incubating cells in glucose-free DMEM medium under hypoxic conditions (95% N_2_ and 5% CO_2_) for 2 h, followed by reoxygenation for 6 h or 24 h in normal medium. TMP was added at the onset of reoxygenation.

### 2.7. Gene Silencing and Plasmid Transfection

HT22 cells were transfected with Parkin siRNA, YAP siRNA, or negative control (NC) siRNA (50 nM; RiboBio, Guangzhou, China) using the riboFECT CP Transfection Kit (RiboBio; catalog no. C10511-05). For rescue experiments, cells were transfected with a *Park2* overexpression plasmid (pCDH-*Park2*) or empty vector (pCDH-Vector) using Lipofectamine 3000 (Invitrogen, Carlsbad, CA, USA; catalog no. L3000001). Briefly, the full-length *Park2* coding sequence (1464 bp) was cloned into the pCDH-PURO vector between the NheI and BamHI restriction sites to generate the pCDH-*Park2* plasmid, while the corresponding empty pCDH vector was used as the control. The inserted sequence was verified by Sanger sequencing using CMV-F and pCDH-R primers, confirming the correct insertion of the *Park2* coding sequence. After 24 h of transfection, cells were subjected to OGD/R.

### 2.8. Pharmacological Treatments

Mitophagy and YAP activity were inhibited by Mdivi-1 (25 μM; MedChemExpress, Monmouth Junction, NJ, USA; catalog no. HY-15886) and Verteporfin (10 μM), respectively, applied 6 h before OGD and maintained during reoxygenation. Bafilomycin A1 (Yeasen Biotechnology, Shanghai, China; catalog no. 53768ES76) was added at a final concentration of 50 nM at 4 h after reoxygenation.

### 2.9. RNA Extraction and RT–qPCR

Total RNA was extracted with TRIzol reagent (Invitrogen; catalog no. 15596026CN). Complementary DNA (cDNA) was synthesized from 1 μg of total RNA using the PrimeScript RT Kit (TaKaRa, Shiga, Japan; catalog no. RR036A). Quantitative real-time PCR was performed on a CFX96 Real-Time PCR System (Bio-Rad, Hercules, CA, USA) using TB Green^®^ Premix (TaKaRa; catalog no. RR420A). The 20 μL reaction system contained cDNA, primers and premix. The PCR program was as follows: 95 °C for 30 s, followed by 40 cycles of 95 °C for 5 s and 60 °C for 30 s. β-actin was used as the internal reference. Relative gene expression was calculated using the 2^−ΔΔCt^ method, with triplicate experiments. The primer sequences used were as follows (*Park2*): Forward: GAGCACACCCAACCTCAGACAAG; Reverse: ACGGAGGCACCAGATGACAGAG.

### 2.10. Western Blot Analysis

Total proteins were extracted using RIPA lysis buffer and quantified with a BCA assay kit (Thermo Fisher Scientific, Waltham, MA, USA; catalog no. 23227). Proteins were separated by SDS-PAGE and transferred onto PVDF membranes. After blocking, the blots were incubated with primary antibodies against Parkin (1:1000; Affinity Biosciences, Changzhou, China; catalog no. AF0235), LC3 (1:1000; Proteintech, Wuhan, China; catalog no. 14600-1-AP), p62 (1:1000; Proteintech; catalog no. 18420-1-AP), YAP (1:1000; Proteintech; catalog no. 13584-1-AP), p-YAP (Ser127; 1:1000; Affinity Biosciences; catalog no. AF3328), and β-actin (1:5000; Proteintech; catalog no. 66009-1-Ig) overnight at 4 °C, followed by a 1 h treatment with HRP-conjugated secondary antibodies (1:5000; Thermo Fisher Scientific). Blots were subjected to ECL detection reagents.

### 2.11. Immunofluorescence Staining

Mice were perfused with saline followed by 4% paraformaldehyde. Brains were post-fixed, cryoprotected in sucrose, and sectioned coronally (20 μm). HT22 cells were cultured in confocal dishes and subjected to OGD for 2 h followed by 6 h of reperfusion. Brain sections and cells were permeabilized with 0.3% Triton X-100, blocked with 5% bovine serum albumin, and incubated overnight at 4 °C with primary antibodies against NeuN (1:500; Huabio, Woburn, MA, USA; catalog no. HA601397), LC3 (1:500; Proteintech; catalog no. 14600-1-AP), TOM20 (1:500; Proteintech; catalog no. 66777-1-Ig), Parkin (1:500; Proteintech; catalog no. 14060-1-AP), β-Tubulin (1:500; Yeasen; catalog no. 30301ES60), or YAP (1:500; Proteintech; catalog no. 13584-1-AP). After washing, sections and cells were incubated with the appropriate FITC- or Cy3-conjugated secondary antibodies (1:1000; Thermo Fisher Scientific) for 1 h at room temperature in the dark. Nuclei were counterstained with DAPI (1:1000; Thermo Fisher Scientific) for 10 min. Fluorescence images were acquired using a confocal laser scanning microscope (LSM 880, Zeiss, Oberkochen, Germany). Colocalization was evaluated using Pearson’s correlation coefficients, and fluorescence intensity distribution was analyzed by line-scan profiling.

### 2.12. Transmission Electron Microscopy (TEM)

After OGD for 2 h followed by 6 h of reperfusion, cells were fixed with glutaraldehyde and osmium tetroxide, embedded, ultrathin-sectioned, and stained with uranyl acetate and lead citrate before ultrastructural observation by TEM (H-7500, Hitachi, Tokyo, Japan). Mitochondrial and endoplasmic reticulum (ER) lengths were quantified from TEM images using ImageJ 1.54 software (NIH, Bethesda, MD, USA). For mitochondrial measurements, the maximal longitudinal axis of individual mitochondria was measured manually using the line tool. For ER analysis, the length of continuous ER segments visible in the cytoplasm was measured. At least 50 mitochondria and 40 ER profiles were analyzed per group from three independent experiments. All measurements were performed in a blinded manner to avoid bias.

### 2.13. RNA Sequencing (RNA-Seq)

Total RNA was extracted from viable HT22 cells in the OGD/R group and the TMP-treated group (100 μM) using TRIzol reagent. RNA quality and integrity were evaluated prior to library preparation. Sequencing libraries were constructed and subjected to paired-end sequencing (2 × 150 bp) on an Illumina NovaSeq 6000 platform (LC-Bio Technology, Hangzhou, China). Differentially expressed genes (DEGs) were identified using DESeq2. To control for multiple testing, *p*-values were adjusted using the Benjamini–Hochberg false discovery rate (FDR) method. Genes with |log2 fold change| ≥ 1 and FDR < 0.05 were considered significantly differentially expressed. A volcano plot was generated to visualize the distribution of DEGs. Kyoto Encyclopedia of Genes and Genomes (KEGG) pathway enrichment analysis was performed to identify significantly enriched signaling pathways associated with TMP treatment.

### 2.14. Cell Viability Assay (CCK-8)

Cell viability was assessed using the CCK-8 assay (Yeasen, Shanghai, China; catalog no. 40203ES80). After OGD 2 h/R 24 h, cells were incubated with CCK-8 solution for 2 h at 37 °C, and the optical density (OD) was measured using a microplate reader (BioTek, Winooski, VT, USA) at 450 nm. Cell viability was calculated as follows: viability (%) = (experimental absorbance/control absorbance) × 100%.

### 2.15. Mitochondrial Reactive Oxygen Species (ROS) Measurement

Mitochondrial ROS was assessed using MitoSOX Red (Yeasen, Shanghai, China; catalog no. 40778ES50). Following OGD 2 h/R 6 h, the HT22 cells were stained with MitoSOX (5 µM) for 10 min at 37 °C without light. Hoechst 33342 (1:1000; Thermo Fisher Scientific; catalog no. R37605) was used to stain the nucleus for 10 min at RT. Cells were visualized using an LSM 880 confocal microscope.

### 2.16. Mitochondrial Membrane Potential (JC-1) Assay

After OGD 2 h/R 6 h, cells were incubated with JC-1 working solution (Beyotime Biotechnology, Shanghai, China; catalog no. C2006) at 37 °C for 15 min. Cells were then centrifuged and resuspended in buffer, and immediately analyzed using a CytoFLEX S flow cytometer (Beckman Coulter, Brea, CA, USA). JC-1 aggregates and monomers were detected in the FL2 and FL1 channels, respectively.

### 2.17. Apoptosis Analysis (Annexin V-FITC/PI)

Apoptosis was assessed using an Annexin V-FITC/PI apoptosis detection kit (Yeasen, Shanghai, China; catalog no. 40302ES60). After OGD for 2 h followed by 24 h of reperfusion, HT22 cells were harvested and stained with Annexin V-FITC and PI according to the manufacturer’s instructions. Samples were then analyzed using a CytoFLEX S flow cytometer (Beckman Coulter, Brea, CA, USA).

### 2.18. Quantification of Mitochondrial DNA Content

Mitochondrial DNA (mtDNA) content was quantified by measuring the mtDNA/nDNA ratio using quantitative PCR. Genomic DNA was extracted from mouse brain tissues using the Ezup Column Animal Genomic DNA Extraction Kit (Sangon Biotech, Shanghai, China; catalog no. B518251) according to the manufacturer’s instructions. The relative mitochondrial DNA content was determined by amplifying mitochondrial genes mt-Nd1 and mt-MitoF1, normalized to the nuclear gene B2m. Quantitative PCR was performed using TB Green Premix (TaKaRa, Shiga, Japan; catalog no. RR420A) on a Real-Time PCR System. The primer sequences were as follows: B2m-F: ATGGGAAGCCGAACATACTG; B2m-R: CAGTCTCAGTGGGGGTGAAT. mt-Nd1-F: TACAACCATTTGCAGACGCC; mt-Nd1-R: TGTGAGTGATAGGGTAGGTGC. mt-MitoF1-F: CTAGAAACCCCGAAACCAAA; mt-MitoR1-R: CCAGCTATCACCAAGCTCGT. The relative mtDNA content was calculated as the ratio of mtDNA to nuclear DNA (mtDNA/nDNA).

### 2.19. Statistical Analysis

Data were represented as mean ± SEM. GraphPad Prism 10 software was used for data analysis. Differences between the two groups were assessed by unpaired t-test. One-way ANOVA followed by Tukey’s post hoc test was used to assess the significance among three or more groups. Statistical significance was established at *p* < 0.05.

## 3. Results

### 3.1. Tetramethylpyrazine (TMP) Alleviates Cerebral Ischemia/Reperfusion Injury (CI/RI) In Vivo

**Figure 1:**
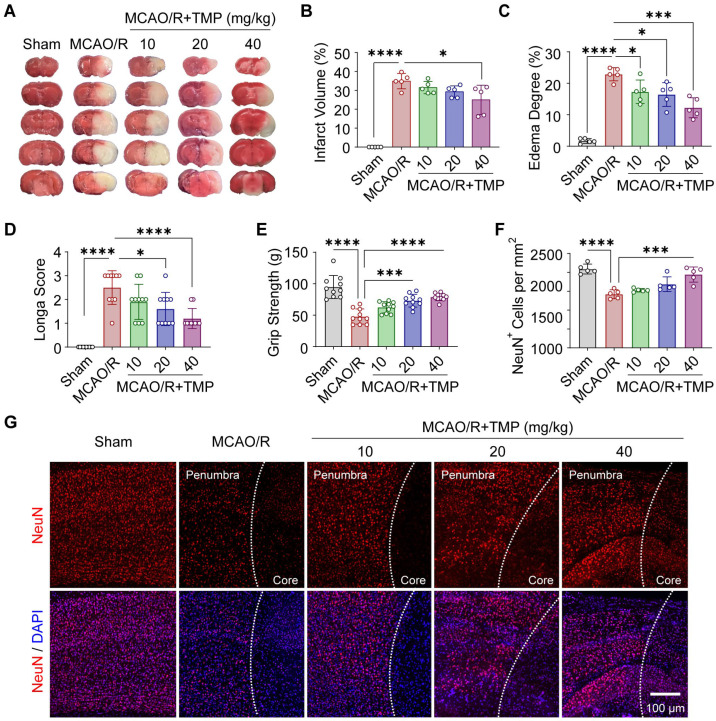
TMP alleviates cerebral ischemia/reperfusion injury after the MCAO/R model in mice. (**A**) Representative TTC-stained coronal brain sections showing infarct areas (white) and viable tissue (red) in Sham, MCAO/R, and MCAO/R + TMP-treated (10, 20, and 40 mg/kg) mice. (**B**,**C**) Quantification of infarct volume and cerebral edema. n = 5 per group. (**D**,**E**) Neurological deficit scores and grip strength measurements at 24 h after reperfusion. n = 10 per group. (**F**) Quantification of NeuN^+^ neuronal density. n = 5 per group. (**G**) Representative immunofluorescence images of NeuN^+^ neurons (red) in the cortical penumbra. Nuclei were counterstained with DAPI (blue). Scale bar: 100 μm. Data are presented as mean ± SEM. * *p* < 0.05, *** *p* < 0.001, **** *p* < 0.0001.

The neuroprotective effects of TMP against CI/RI were evaluated using the MCAO/R mouse model, in which cerebral ischemia was induced for 60 min followed by 24 h of reperfusion. TMP was administered intraperitoneally at the onset of reperfusion. TTC staining revealed extensive pale infarct regions in MCAO/R mice, whereas TMP treatment (40 mg/kg) following MCAO/R markedly preserved tissue viability, resulting in substantially smaller infarct areas (Figure 1A,B) and attenuated hemispheric swelling (Figure 1C). MCAO/R mice displayed severe neurological deficits and markedly reduced grip strength, both of which were significantly improved by TMP administration following MCAO/R (Figure 1D,E). Furthermore, immunofluorescence staining for NeuN revealed pronounced neuronal loss in the cortical penumbra of MCAO/R mice. Treatment with TMP at 40 mg/kg following MCAO/R significantly restored neuronal density (Figure 1F,G). Collectively, these findings demonstrate that TMP confers robust neuroprotection during the acute phase of CI/RI.

### 3.2. TMP Enhances Mitophagy in Ischemic Brain Tissue After Reperfusion

**Figure 2:**
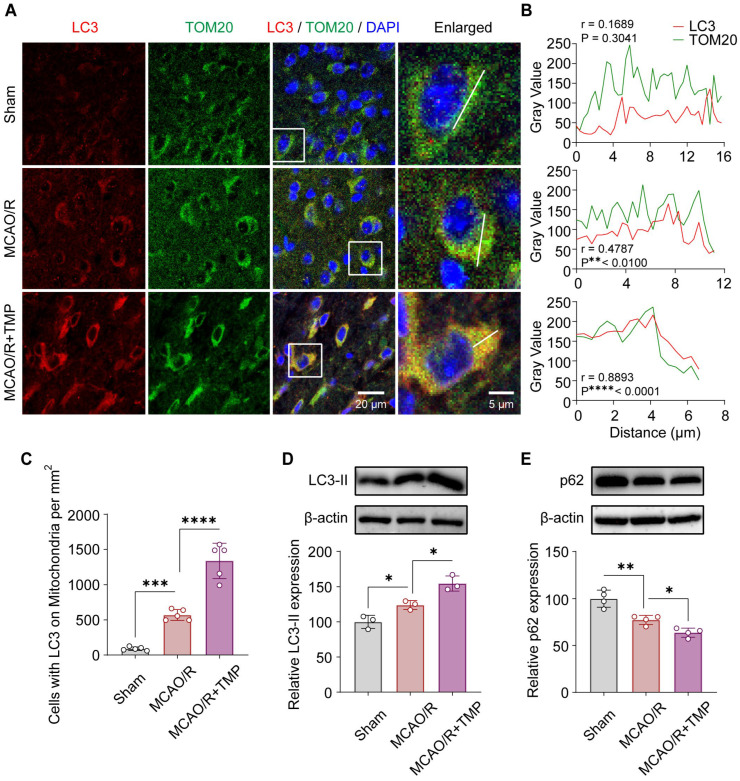
TMP enhances mitophagy in ischemic brain tissue after reperfusion. (**A**) Representative immunofluorescence images of LC3 (red) and the mitochondrial marker TOM20 (green) in the cortical ischemic penumbra of Sham, MCAO/R, and MCAO/R + TMP-treated (40 mg/kg) mice. Nuclei were counterstained with DAPI (blue). Scale bars = 20 μm and 5 μm. (**B**) Line-scan intensity profiles of LC3 and TOM20 fluorescence along the indicated lines in panel (**A**). Pearson’s correlation coefficients (r) and *p* values indicate the degree of LC3–mitochondria colocalization. (**C**) Quantification of cells containing LC3-positive mitochondria in the ischemic penumbra. n = 5 per group. (**D**) Representative Western blot and densitometric analysis of LC3-II expression, normalized to β-actin. n = 3 per group. (**E**) Representative Western blot and densitometric analysis of p62 expression, normalized to β-actin. n = 4 per group. Data are presented as mean ± SEM. * *p* < 0.05, ** *p* < 0.01, *** *p* < 0.001, **** *p* < 0.0001. Original images of Western blotting can be found in Supplementary Materials.

To determine whether TMP promotes mitophagy in vivo, the subcellular localization of the autophagy marker LC3 relative to mitochondria labeled by TOM20 was examined in the ischemic penumbra. In the Sham group, LC3 signals were weak and diffusely distributed, showing minimal colocalization with TOM20. Following MCAO/R, LC3 immunoreactivity was modestly increased but remained largely dispersed, with limited overlap with mitochondrial marker protein TOM20. In contrast, TMP-treated (40 mg/kg) following MCAO/R exhibited robust punctate LC3 signals that extensively colocalized with TOM20, forming prominent yellow puncta, indicative of enhanced autophagosome–mitochondria association (Figure 2A). Line-scan analysis further revealed weak LC3/TOM20 correlation in Sham, a moderate increase after MCAO/R, and a strong positive correlation following MCAO/R + TMP treatment (Figure 2B), accompanied by a significant increase in LC3-positive mitochondria-containing cells (Figure 2C). Western blot analysis confirmed increased LC3-II and decreased p62 levels after MCAO/R, both of which were further modulated by TMP, indicating enhanced mitophagy in ischemic brain tissue (Figure 2D,E). To further evaluate mitochondrial content, the mtDNA/nDNA ratio was measured in mouse brain tissues by qPCR using mitochondrial genes (mt-Nd1 and mt-MitoF1) normalized to the nuclear gene B2m. MCAO/R significantly increased the mtDNA/nDNA ratio, whereas TMP treatment markedly reduced mitochondrial DNA levels, indicating enhanced mitochondrial clearance (Supplementary Figure S1). Collectively, these results indicate that TMP markedly enhances mitophagy in ischemic brain tissue following cerebral ischemia/reperfusion injury.

### 3.3. TMP Preserves Mitochondrial Function and Promotes Mitophagy-Dependent Cytoprotection After OGD/R in HT22 Cells

**Figure 3:**
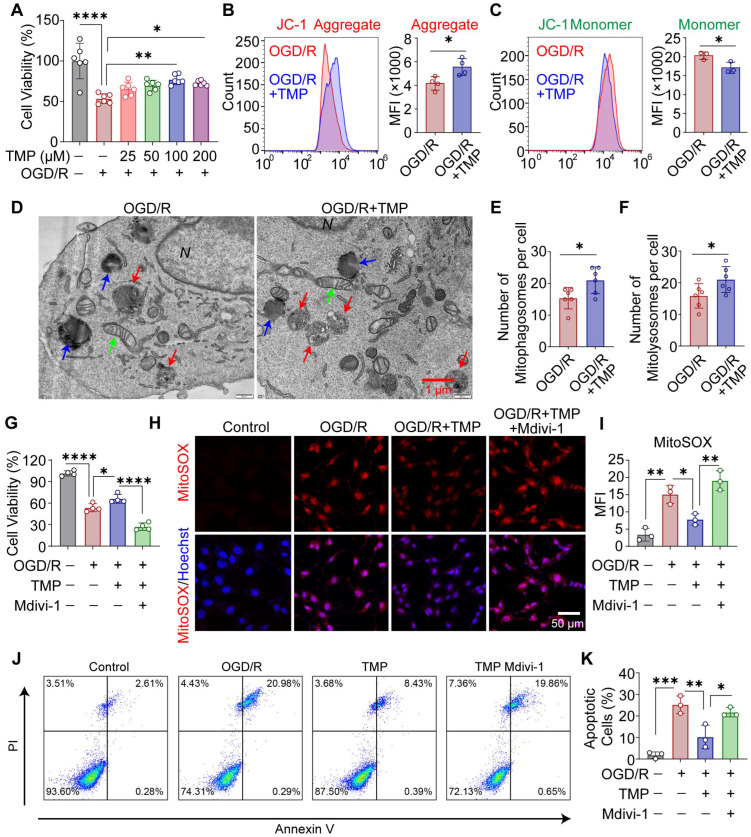
TMP preserves mitochondrial function and promotes mitophagy-dependent cytoprotection after OGD/R in HT22 cells. (**A**) Cell viability of HT22 cells subjected to OGD/R and treated with increasing concentrations of TMP after OGD/R. n = 6 independent experiments. (**B**) Representative flow cytometry histograms and quantification of JC-1 aggregates in OGD/R and TMP-treated (100 μM) cells. n = 4 independent experiments. (**C**) Representative flow cytometry histograms and quantification of JC-1 monomers. n = 3 independent experiments. (**D**) Representative TEM images showing mitochondrial ultrastructure in OGD/R and TMP-treated (100 μM) cells. Green arrows indicate normal mitochondria, red arrows indicate mitophagosomes, and blue arrows indicate mitolysosomes. N, nucleus. Scale bar = 1 μm. (**E**,**F**) Quantification of the number of mitophagosomes and mitolysosomes per cell. n = 6 cells per group from three independent experiments. (**G**) Cell viability assessed by CCK-8 in control, OGD/R, TMP-treated (100 μM), and TMP + Mdivi-1-treated HT22 cells. n = 4 independent experiments. (**H**) Representative MitoSOX staining images showing mitochondrial ROS levels. Nuclei were counterstained with Hoechst. Scale bar = 50 μm. (**I**) Quantification of MitoSOX fluorescence intensity. n = 3 independent experiments. (**J**) Representative flow cytometry plots of Annexin V–FITC/PI staining. (**K**) Quantification of apoptotic (FITC^+^) cells determined by Annexin V–FITC/PI flow cytometric analysis. n = 3 independent experiments. Data are presented as mean ± SEM. * *p* < 0.05, ** *p* < 0.01, *** *p* < 0.001, **** *p* < 0.0001.

To evaluate the protective effects of TMP in vitro, HT22 cells were subjected to OGD/R. CCK-8 assays showed that TMP exhibited no detectable cytotoxicity in HT22 cells at concentrations up to 800 μM (Supplementary Figure S2A). Cell viability was markedly reduced following OGD/R, whereas 100 μM TMP treatment after OGD/R produced the most pronounced improvement in cell survival (Figure 3A). Mitochondrial function was assessed by JC-1 staining. TMP treatment after OGD/R significantly increased JC-1 aggregate levels and reduced monomer signals relative to the OGD/R group, indicating partial restoration of mitochondrial membrane potential (Figure 3B,C). Consistently, TMP attenuated OGD/R-induced mitochondrial ROS accumulation, as determined by MitoSOX staining (Supplementary Figure S2B,C). To further evaluate mitochondrial function, intracellular ATP levels were measured to assess cellular energy metabolism. The results showed that OGD/R significantly reduced ATP content, whereas TMP treatment partially restored ATP production (Supplementary Figure S2D). Transmission electron microscopy revealed swollen mitochondria with disrupted cristae after OGD/R, whereas TMP treatment induced abundant mitophagosomes and mitolysosomes containing partially degraded mitochondria, indicative of enhanced mitophagy (Figure 3D–F). TMP also significantly restored mitochondrial and endoplasmic reticulum length, suggesting improved ultrastructural integrity (Supplementary Figure S2E,F).

Since mitophagy is a dynamic process, we next examined whether TMP influences autophagic flux. To this end, the lysosomal inhibitor bafilomycin A1 (BafA1) was used to block autophagosome degradation. Under OGD/R conditions, BafA1 treatment resulted in increased accumulation of LC3-II and p62. Importantly, in the presence of BafA1, TMP further increased LC3-II levels and the LC3-II/LC3-I ratio, accompanied by additional p62 accumulation, suggesting enhanced autophagic flux rather than impaired degradation (Supplementary Figure S3).

To assess the requirement of mitophagy for TMP-mediated protection, mitophagy was inhibited with Mdivi-1. TMP significantly improved cell viability and reduced mitochondrial ROS accumulation after OGD/R, whereas these effects were largely abolished by Mdivi-1 treatment (Figure 3G–I). Consistently, TMP attenuated OGD/R-induced apoptosis, effects that were also reversed by Mdivi-1, as determined by Annexin V/PI flow cytometry (Figure 3J,K).

### 3.4. TMP Promotes Parkin-Dependent Mitophagy After Cerebral Ischemia/Reperfusion

**Figure 4:**
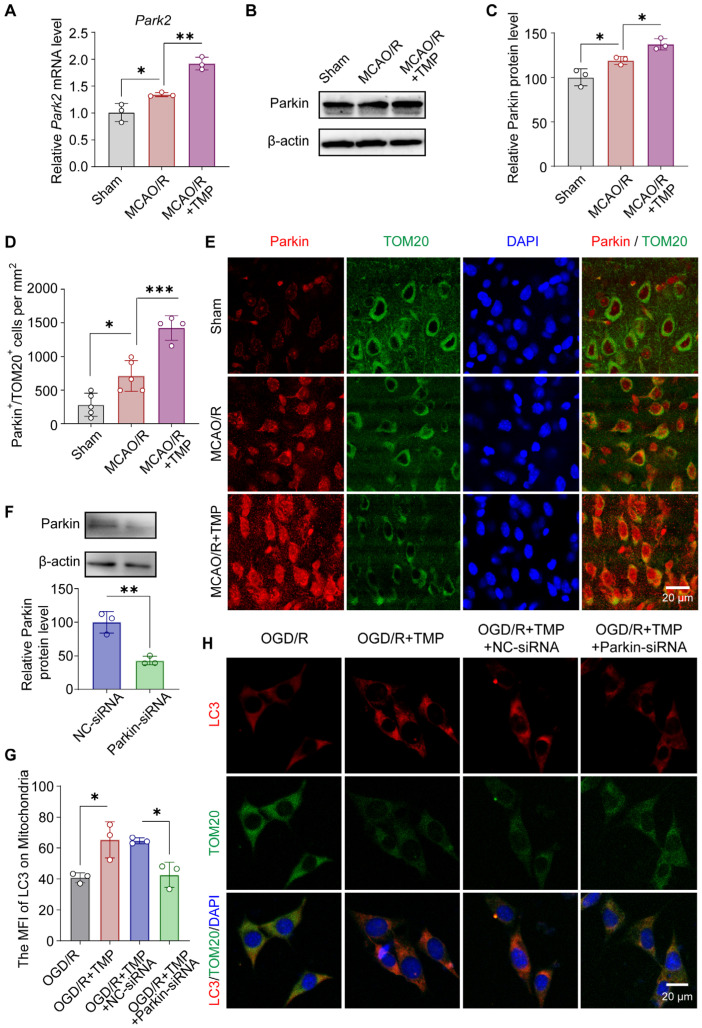
TMP promotes Parkin-dependent mitophagy after cerebral ischemia/reperfusion. (**A**) *Park2* mRNA expression in Sham, MCAO/R, and TMP (40 mg/kg) groups. n = 3 per group. (**B**,**C**) Representative Western blot images and quantification of Parkin protein levels. Normalized to β-actin. n = 3 per group. (**D**) Representative immunofluorescence images showing Parkin (red), TOM20 (green), and nuclei (blue). Scale bar: 20 μm. (**E**) Quantification of Parkin/TOM20 positive cells per mm2. n = 4–5 per group. (**F**) Western blot images and quantification of Parkin protein levels to verify the silencing efficiency of siRNA. Normalized to β-actin. n = 3 per group. (**G**) Quantification of the mean fluorescence intensity (MFI) of LC3 colocalized with TOM20. n = 3 independent experiments. (**H**) Representative immunofluorescence images showing LC3 (red) and TOM20 (green) localization in OGD/R-injured HT22 cells treated with 100 μM TMP, TMP plus negative control siRNA (NC-siRNA), or TMP plus Parkin siRNA. Nuclei were counterstained with DAPI (blue). Scale bar = 20 μm. Data are presented as mean ± SEM. * *p* < 0.05, ** *p* < 0.01, *** *p* < 0.001.

To determine whether TMP-induced mitophagy depends on Parkin, Parkin expression and its mitochondrial localization were examined following cerebral ischemia/reperfusion. *Park2* mRNA and Parkin protein levels were increased after MCAO/R and were further elevated by TMP treatment (Figure 4A–C). Immunofluorescence analysis revealed enhanced Parkin translocation to mitochondria in TMP-treated mice, as indicated by increased Parkin/TOM20 colocalization in the peri-infarct cortex (Figure 4D–E). To assess the requirement of Parkin for TMP-induced mitophagy, Parkin was selectively silenced by siRNA in HT22 cells. The silencing efficiency of siRNA was verified by Western blot (Figure 4F). TMP markedly enhanced LC3 recruitment to mitochondria in the presence of negative control (NC) siRNA, whereas this effect was significantly attenuated by Parkin knockdown (Figure 4G,H). In parallel, TMP-induced suppression of OGD/R-triggered mitochondrial ROS accumulation was abolished by Parkin silencing, resulting in elevated ROS levels (Supplementary Figure S4). These results indicate that Parkin is required for TMP-induced mitophagy and the associated mitochondrial protective effects.

### 3.5. TMP Modulates YAP Activity and Nuclear Localization After Cerebral Ischemia/Reperfusion

**Figure 5:**
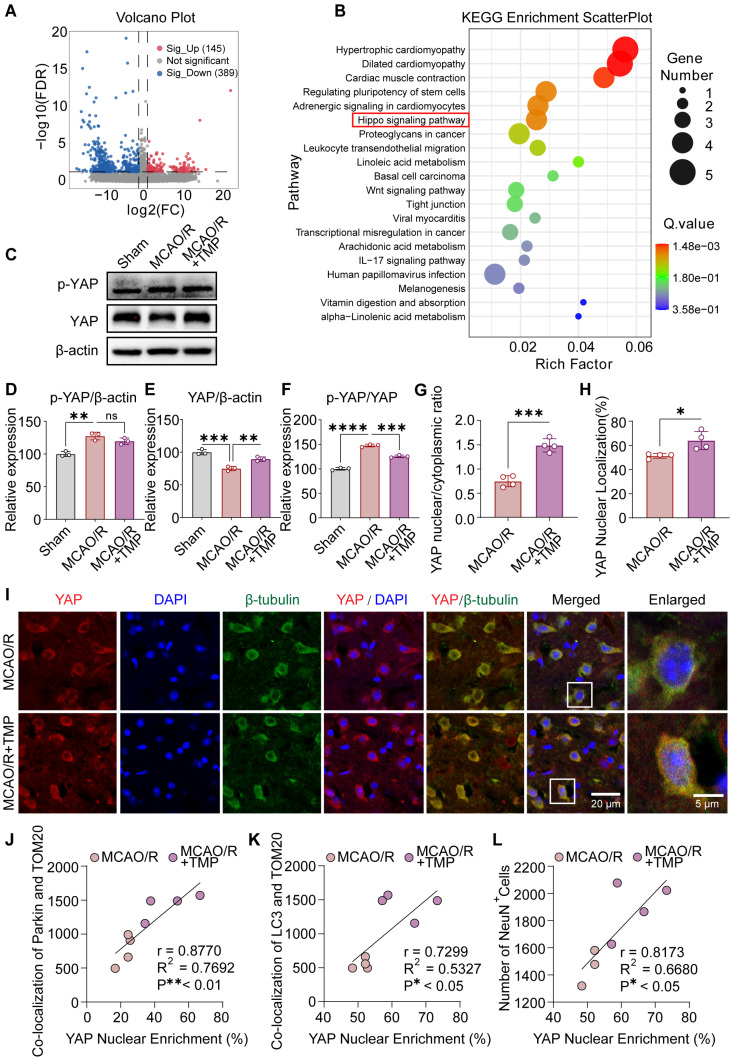
TMP modulates YAP activity and nuclear localization after cerebral ischemia/reperfusion. (**A**) Volcano plot showing differentially expressed genes following TMP treatment. Significant DEGs were defined as |log2FC| ≥ 1 and FDR < 0.05. (**B**) KEGG pathway enrichment analysis of differentially expressed genes. (**C**) Representative Western blot images of total YAP and p-YAP (Ser127) expression in Sham, MCAO/R, and TMP-treated (40 mg/kg) groups. (**D**–**F**) Quantitative analysis of p-YAP/β-actin, total YAP/β-actin, and p-YAP/YAP ratios. n = 3 per group. (**G**) Quantification of the YAP nuclear/cytoplasmic ratio. n = 4 per group. (**H**) Quantification of the percentage of cells exhibiting nuclear YAP localization. n = 4 per group. (**I**) Representative immunofluorescence images showing YAP (red), β-Tubulin (green), and DAPI (blue). Scale bars = 20 μm and 5 μm. (**J**–**L**) Correlation analyses between YAP nuclear localization and Parkin recruitment to mitochondria, LC3/TOM20 colocalization, and NeuN^+^ neuronal density. Data are presented as mean ± SEM. ns, not significant; * *p* < 0.05, ** *p* < 0.01, *** *p* < 0.001, **** *p* < 0.0001.

To explore upstream mechanisms underlying TMP-induced mitophagy, transcriptomic analysis was performed. Differentially expressed genes were visualized by a volcano plot (Figure 5A), and KEGG enrichment analysis identified the Hippo signaling pathway as significantly enriched following TMP treatment (Figure 5B). Given that YAP is a central effector of Hippo signaling, its activity was further examined. Western blot analysis showed that MCAO/R markedly increased p-YAP (Ser127) levels and disrupted YAP expression, whereas TMP treatment partially normalized YAP/β-actin and p-YAP/YAP ratios (Figure 5C–F).

Consistent with the Western blot results, immunofluorescence analysis further showed that TMP treatment markedly promoted YAP nuclear translocation following MCAO/R injury. Quantitative analysis revealed that the nuclear/cytoplasmic ratio of YAP and the percentage of nuclear YAP localization were significantly increased in the MCAO/R + TMP group compared with the MCAO/R group (Figure 5G–I). Notably, YAP nuclear enrichment exhibited a strong positive correlation with Parkin recruitment to mitochondria, LC3/TOM20 colocalization, and NeuN^+^ neuronal survival (Figure 5J–L). Collectively, these results indicate that TMP restores YAP activity and promotes its nuclear localization after cerebral ischemia/reperfusion, potentially contributing to the modulation of Parkin-associated mitophagy.

### 3.6. YAP Activity Is Required for TMP-Induced Mitophagy Activation and Parkin Upregulation

**Figure 6:**
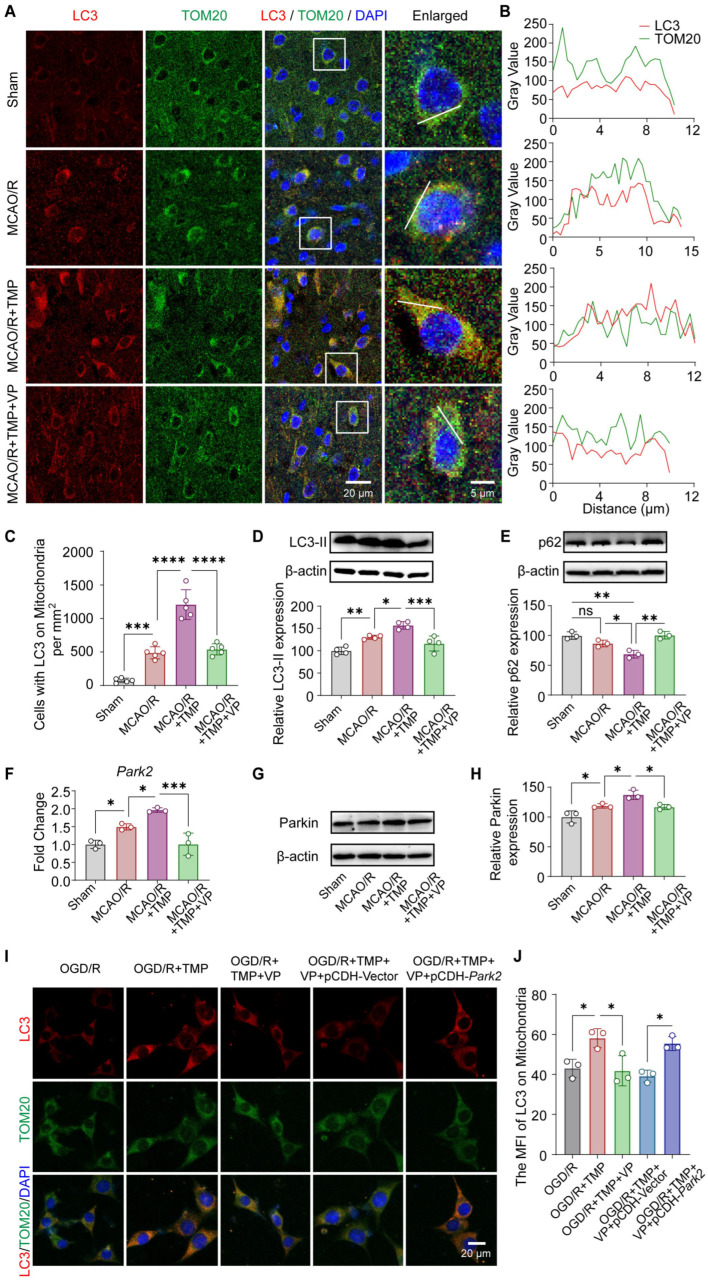
YAP activity is required for TMP-induced Parkin upregulation and mitophagy activation. (**A**) Representative immunofluorescence images showing LC3 (red), TOM20 (green), and nuclei (DAPI, blue) in the cortical penumbra. Scale bars: 20 μm and 5 μm (enlarged images). (**B**) Line-scan intensity profile analysis of LC3 and TOM20 signals along the indicated lines in enlarged images. (**C**) Quantification of LC3-positive mitochondria per mm^2^ in different treatment groups. n = 5 per group. (**D**,**E**) Representative Western blot images and quantitative analysis of LC3-II and p62 protein levels normalized to β-actin. n = 3–4 per group. (**F**) Quantitative PCR analysis of *Park2* mRNA expression in Sham, MCAO/R, TMP-treated (40 mg/kg), and TMP + VP groups. n = 3 per group. (**G**,**H**) Representative Western blot images and quantitative analysis of Parkin protein expression, with β-actin as a loading control. n = 3 per group. (**I**) Representative immunofluorescence images showing LC3 (red), TOM20 (green), and DAPI (blue) in OGD/R-injured neurons treated with TMP (100 μM), TMP + VP, TMP + VP + pCDH-Vector, or TMP + VP + pCDH-*Park2*. Scale bars: 20 μm. (**J**) Quantification of LC3 recruitment to mitochondria based on mean fluorescence intensity (MFI). n = 3 independent experiments. Data are presented as mean ± SEM. ns, not significant; * *p* < 0.05, ** *p* < 0.01, *** *p* < 0.001, **** *p* < 0.0001. Original images of Western blotting can be found in Supplementary Materials.

To further delineate the role of YAP in TMP-induced mitophagy activation and Parkin upregulation, the YAP inhibitor Verteporfin (VP) was employed. Immunofluorescence quantification showed that LC3/TOM20 colocalization was enhanced by TMP treatment, which was markedly reversed by VP administration, as confirmed by line-scan intensity profile analysis and quantitative analysis (Figure 6A–C). At the protein level, TMP markedly increased LC3-II accumulation and reduced p62 expression. These effects were significantly attenuated by VP treatment (Figure 6D,E), suggesting that YAP inhibition compromises TMP-induced mitophagy.

Moreover, TMP increased *Park2* mRNA and Parkin protein expression after MCAO/R, effects that were significantly attenuated by VP treatment (Figure 6F–H), suggesting that YAP is involved in TMP-induced upregulation of Parkin. To determine whether Parkin acts downstream of YAP in this process, rescue experiments were performed by overexpressing *Park2* in the presence of VP. Immunofluorescence analysis showed that *Park2* overexpression (pCDH-*Park2*) significantly restored LC3 recruitment to mitochondria compared with the empty vector control (pCDH-Vector) (Figure 6I,J). The overexpression efficiency of Parkin was verified by Western blot, as shown in Supplementary Figure S5. To specifically silence YAP expression, HT22 cells were transfected with YAP-siRNA. YAP knockdown significantly reduced TMP-induced Parkin expression and LC3 localization on mitochondria, suggesting that YAP is required for TMP-mediated mitophagy (Supplementary Figure S6). Collectively, these results indicate that YAP activity is required for TMP-induced Parkin upregulation and subsequent activation of mitophagy following ischemic injury.

## 4. Discussion

In the present study, we show that tetramethylpyrazine (TMP) protects against cerebral ischemia/reperfusion (CI/RI) injury by enhancing mitophagy and preserving mitochondrial homeostasis in vivo and in vitro. Notably, we identify Yes-associated protein (YAP) as an upstream regulator of Parkin-dependent mitophagy, revealing a YAP–Parkin axis underlying TMP-mediated neuroprotection under ischemic stress.

Mitophagy, a selective form of autophagy responsible for the removal of damaged mitochondria, plays a pivotal role in mitochondrial quality control under stress conditions [8,13,25]. Growing evidence suggests that insufficient or dysregulated activation of mitophagy can be detrimental, whereas appropriately enhanced mitophagy may confer neuroprotection in CI/RI [8,26]. TMP has been used in the treatment of cerebrovascular disorders and exhibits neuroprotective effects under ischemic conditions [16,17,18]. Notably, previous studies have suggested that TMP modulates mitophagic activity in a tissue- and injury-specific manner [27,28]. These context-dependent results provided further rationale for examining how TMP regulates mitophagy in the setting of cerebral ischemia/reperfusion injury. Our data demonstrate that TMP enhances mitophagic activity, reflected by increased LC3 recruitment to mitochondria and accumulation of mitophagy-related vesicles in models. TMP preserves mitochondrial function under ischemic stress, as evidenced by reduced mitochondrial oxidative burden and restoration of mitochondrial membrane potential. Importantly, pharmacological inhibition of mitophagy partially abolished the protective effects of TMP on mitochondrial function and neuronal survival, supporting a functional contribution of mitophagy to TMP-mediated neuroprotection. These observations provide a mechanistic foundation for further exploration of upstream regulators and signaling pathways that govern mitophagy under ischemic stress.

Among the molecular pathways governing mitophagy, Parkin-mediated ubiquitination represents a principal mechanism of mitochondrial quality control in mammals [29,30,31]. In the context of cerebral ischemia/reperfusion injury, accumulating evidence indicates that activation of Parkin-dependent mitophagy alleviates oxidative stress, preserves mitochondrial function, and reduces neuronal injury, thereby extending the therapeutic window after reperfusion [32,33,34]. Consistent with these observations, the present study demonstrates that TMP enhances Parkin expression and facilitates its recruitment to mitochondria following ischemia/reperfusion insult. Importantly, silencing of Parkin significantly attenuated TMP-induced mitophagic activation and abolished its protective effects on mitochondrial integrity, indicating that Parkin is functionally required for TMP-mediated mitophagy. This mechanistic specificity provides a framework for understanding TMP-mediated neuroprotection and raises the question of how Parkin activity is regulated upstream under ischemic conditions.

YAP, a transcriptional co-activator and central effector of the Hippo signaling pathway, has emerged as an important regulator of cellular stress responses, survival, and metabolic adaptation [19]. Notably, emerging evidence from other disease models supports a functional link between YAP signaling and mitophagy regulation [35,36]. Importantly, *Park2*, which encodes Parkin, has been identified as a transcriptional target of YAP in cardiac disease models [37], providing direct evidence that YAP can regulate Parkin expression at the transcriptional level. Our data demonstrate that TMP promotes YAP activation and nuclear localization following ischemia/reperfusion injury, accompanied by increased Parkin expression and enhanced mitophagic activity. Pharmacological inhibition of YAP markedly attenuated TMP-induced Parkin upregulation and mitophagy activation, whereas restoration of Parkin expression rescued mitophagic activity despite YAP inhibition. These findings identify YAP as a critical upstream regulator of Parkin-dependent mitophagy under ischemic stress.

Based on the present findings, we propose a working model in which TMP promotes YAP activation and nuclear translocation, thereby enhancing Parkin-dependent mitophagy and preserving mitochondrial integrity and function during the acute reperfusion phase. From a translational perspective, TMP, as a neuroprotective agent targeting mitochondrial homeostasis, may complement current reperfusion therapies following successful recanalization. Several limitations warrant consideration. The precise transcriptional mechanisms by which YAP regulates Parkin expression remain to be elucidated, and whether sustained modulation of mitophagy contributes to long-term neurological recovery requires further investigation.

## 5. Conclusions

In conclusion, these findings identify a YAP–Parkin–mitophagy axis underlying TMP-mediated neuroprotection. Moreover, our results suggest that mitophagy modulation is a potential therapeutic strategy and extend the role of YAP as a key regulator of mitophagy during cerebral ischemia/reperfusion injury.

## Data Availability

The original contributions presented in this study are included in the article/Supplementary Material. Further inquiries can be directed to the corresponding author(s).

## Supplementary Materials

The following supporting information can be downloaded at: https://www.mdpi.com/article/10.3390/biom16030429/s1, Figure S1: TMP reduces mitochondrial DNA content following MCAO/R injury; Figure S2: TMP exhibits no cytotoxicity and attenuates mitochondrial oxidative stress and structural damage after OGD/R in HT22 cells; Figure S3: TMP enhances autophagic flux in HT22 cells following OGD/R injury; Figure S4: Parkin knockdown abolishes TMP-mediated suppression of mitochondrial ROS after OGD/R in HT22 cells; Figure S5: Verification of Parkin overexpression in HT22 cells; Figure S6: YAP knockdown attenuates TMP-induced Parkin upregulation and mitophagy in HT22 cells after OGD/R. Original images of Western blotting can be found in Supplementary Materials.

## Abbreviations

The following abbreviations are used in this manuscript:

CI/RI	Cerebral ischemia/reperfusion injury
TMP	Tetramethylpyrazine
MCAO/R	Middle cerebral artery occlusion/reperfusion
OGD/R	Oxygen–glucose deprivation/reoxygenation
YAP	Yes-associated protein
siRNA	Small interfering RNA
NC	Negative control
