# Supplementary material for: Mitophagy Activation via the YAP/Parkin Pathway Underlies the Neuroprotective Action of Tetramethylpyrazine in Cerebral Ischemia/Reperfusion Injury
Source: Biomolecules. 2026 Mar 13;16(3):429. doi: 10.3390/biom16030429 (PMC13024434; doi:10.3390/biom16030429)
Supplement: Supplementary file 1 [file biomolecules-16-00429-s001.zip › 20260308-Original Blots_Supplementary File for Review.pdf]

Western blot images with molecular weight markers retained.

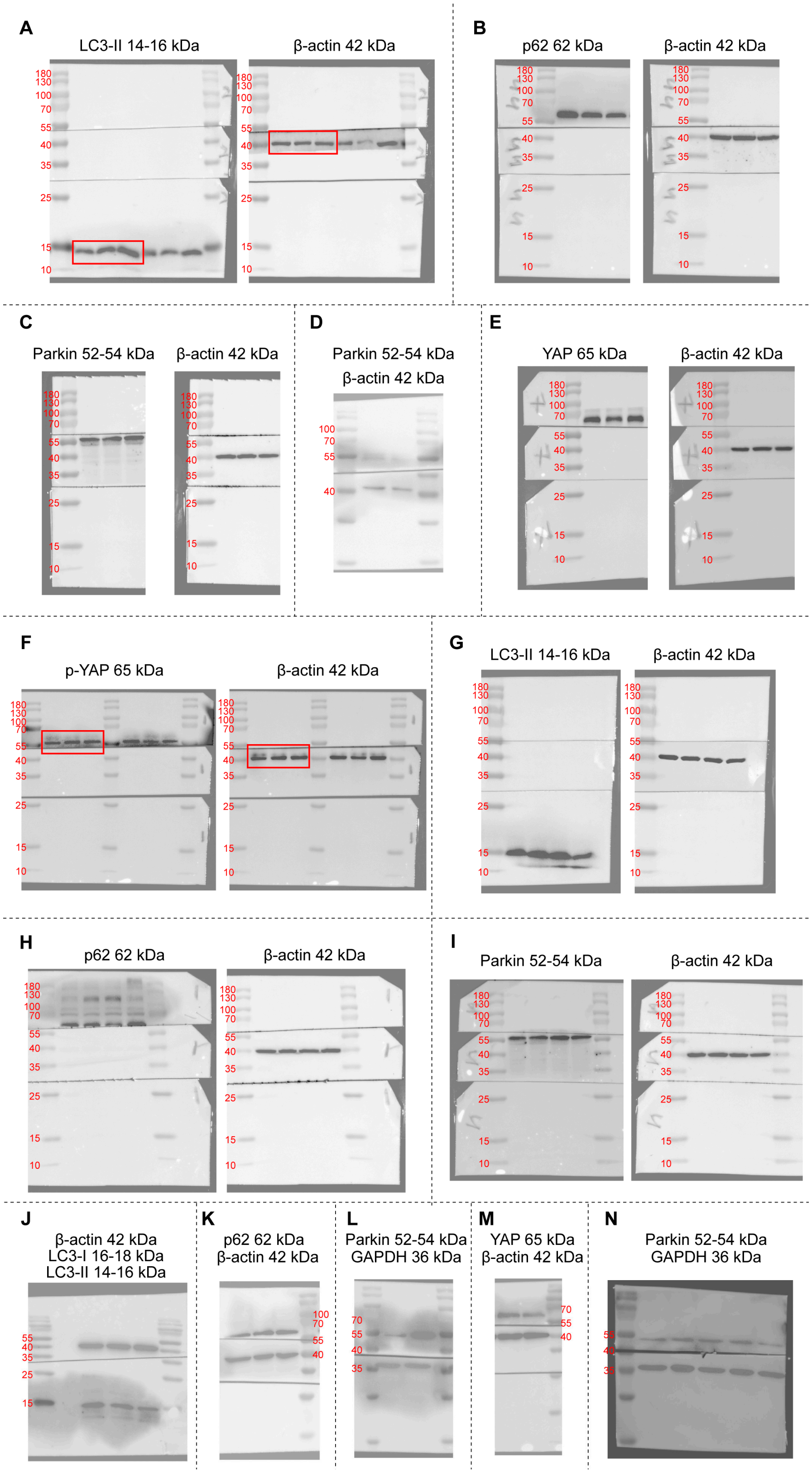

(A) Source western blot image for Figure 2D.

(B) Source western blot image for Figure 2E.

(C) Source western blot image for Figure 4B.

(D) Source western blot image for Figure 4F.

(E-F) Source western blot image for Figure 5C.

(G) Source western blot image for Figure 6D.

(H) Source western blot image for Figure 6E.

(I) Source western blot image for Figure 6G.

(J) Source western blot image for Supplementary Fig. 3A.

(K) Source western blot image for Supplementary Fig. 3E.

(L) Source western blot image for Supplementary Fig. 5A.

(M) Source western blot image for Supplementary Fig. 6A.

(N) Source western blot image for Supplementary Fig. 6B.
