# Supplementary material for: Mitophagy Activation via the YAP/Parkin Pathway Underlies the Neuroprotective Action of Tetramethylpyrazine in Cerebral Ischemia/Reperfusion Injury
Source: Biomolecules. 2026 Mar 13;16(3):429. doi: 10.3390/biom16030429 (PMC13024434; doi:10.3390/biom16030429)
Supplement: Supplementary file 1 [file biomolecules-16-00429-s001.zip › 20260311-Supplementary Figures.pdf]

Supplementary Figures

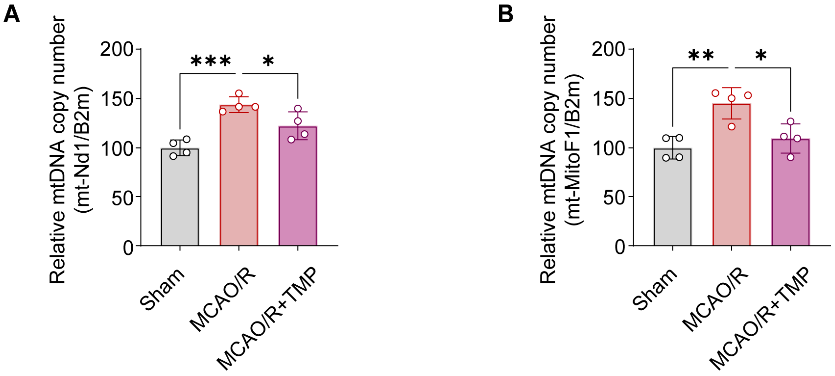

Supplementary Figure S1. TMP reduces mitochondrial DNA content following MCAO/R injury.

(A–B) Quantitative PCR analysis of mitochondrial DNA levels in mouse brain tissues. The mtDNA/nDNA ratio was determined using mitochondrial genes mt-Nd1 (A) and mt-MitoF1 (B), normalized to the nuclear gene B2m. Data are presented as mean ± SD (n = 4). *P < 0.05, **P < 0.01, ***P < 0.001.

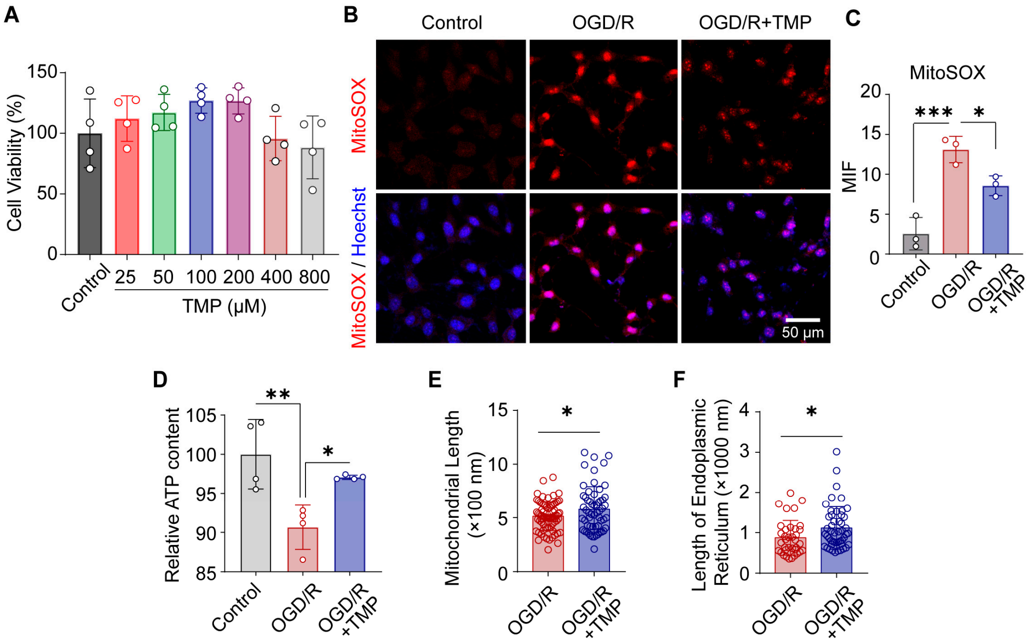

Supplementary Figure S2. TMP exhibits no cytotoxicity and attenuates mitochondrial oxidative stress and structural damage after OGD/R in HT22 cells.

(A) Cell viability of HT22 cells treated with increasing concentrations of TMP, as assessed by CCK-8 assay. (B) Representative MitoSOX staining images showing mitochondrial ROS levels in control, OGD/R, and OGD/R + TMP-treated (100 μM) cells. Nuclei were counterstained with Hoechst. Scale bar = 50 μm. (C) Quantification of MitoSOX fluorescence intensity. n = 3 independent experiments. (D) Intracellular ATP levels in control, OGD/R, and OGD/R + TMP-treated (100 μM) cells. n = 4 independent experiments. (E) Quantification of mitochondrial length (at least 50 mitochondria per group, from three independent experiments). (F) Quantification of endoplasmic reticulum length (at least 40 endoplasmic reticulum profiles per group, from three independent experiments). Data are presented as mean ± SEM. *P < 0.05, **P < 0.01, ***P < 0.001.

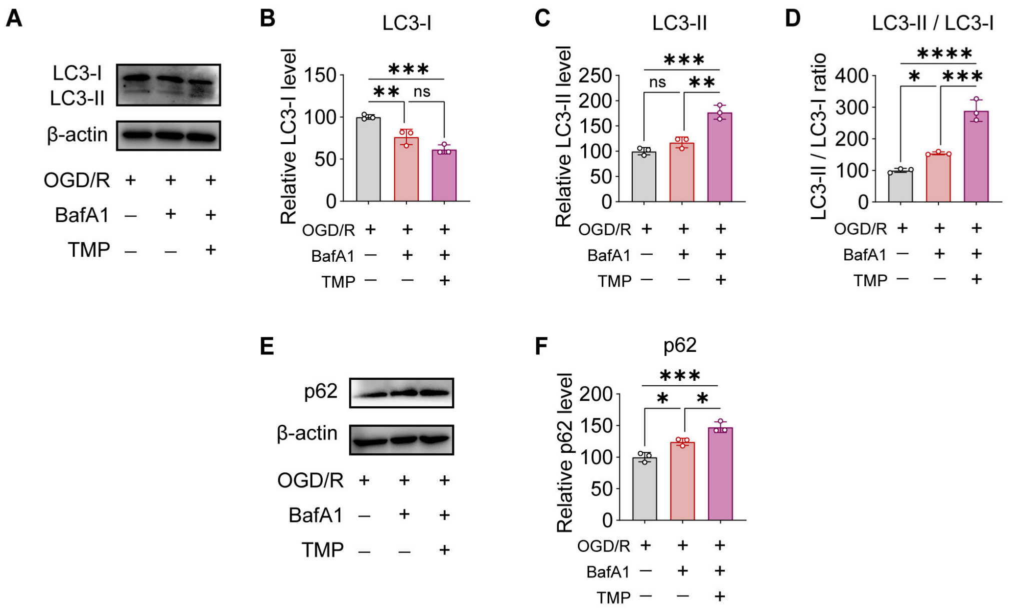

Supplementary Figure S3. TMP enhances autophagic flux in HT22 cells following OGD/R injury.

(A) Representative Western blot images showing the expression of LC3-I and LC3-II in HT22 cells subjected to OGD/R injury with or without Bafilomycin A1 (BafA1) and TMP treatment. (B–C) Quantitative analysis of LC3-I (B) and LC3-II (C) protein levels normalized to β-actin. (D) Quantification of the LC3-II/LC3-I ratio. (E) Representative Western blot images showing p62 expression under the indicated conditions. (F) Quantitative analysis of p62 protein levels normalized to β-actin. Data are presented as mean ± SD (n = 3). *P < 0.05, **P < 0.01, ***P < 0.001, ****P < 0.0001; ns, not significant.

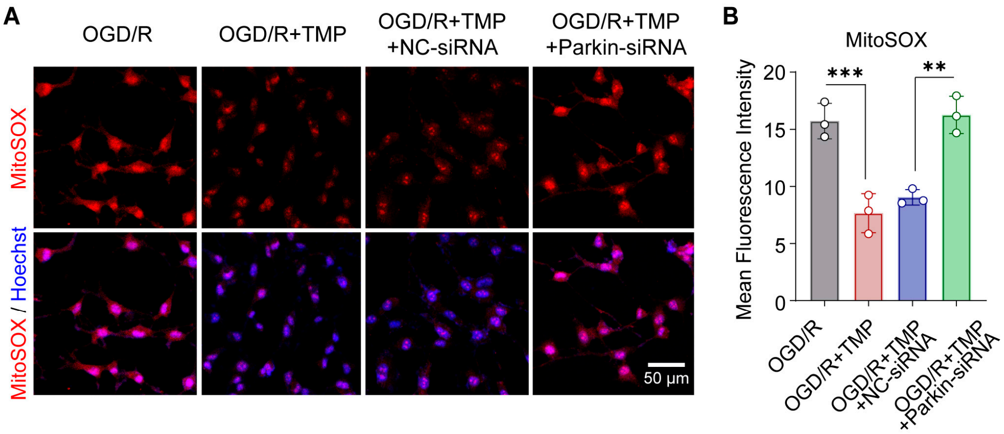

Supplementary Figure S4. Parkin knockdown abolishes TMP-mediated suppression of mitochondrial ROS after OGD/R in HT22 cells.

(A) Representative MitoSOX staining images showing mitochondrial ROS levels in OGD/R-injured HT22 cells treated with 100 μM TMP, TMP plus negative control siRNA (NC-siRNA), or TMP plus Parkin siRNA. Nuclei were counterstained with Hoechst. Scale bar = 50 μm. (B) Quantification of MitoSOX fluorescence intensity. n = 3 independent experiments. Data are presented as mean ± SEM. **P < 0.01, ***P < 0.001.

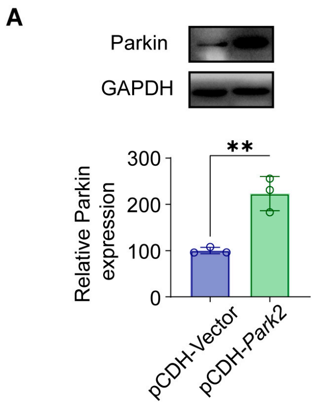

Supplementary Figure S5. Verification of Parkin overexpression in HT22 cells.

(A) Representative Western blot images showing Parkin protein levels in HT22 cells transiently transfected with pCDH-Parkin or empty pCDH vector. GAPDH was used as the loading control. n = 3 independent experiments. Data are presented as mean ± SEM. **P < 0.01.

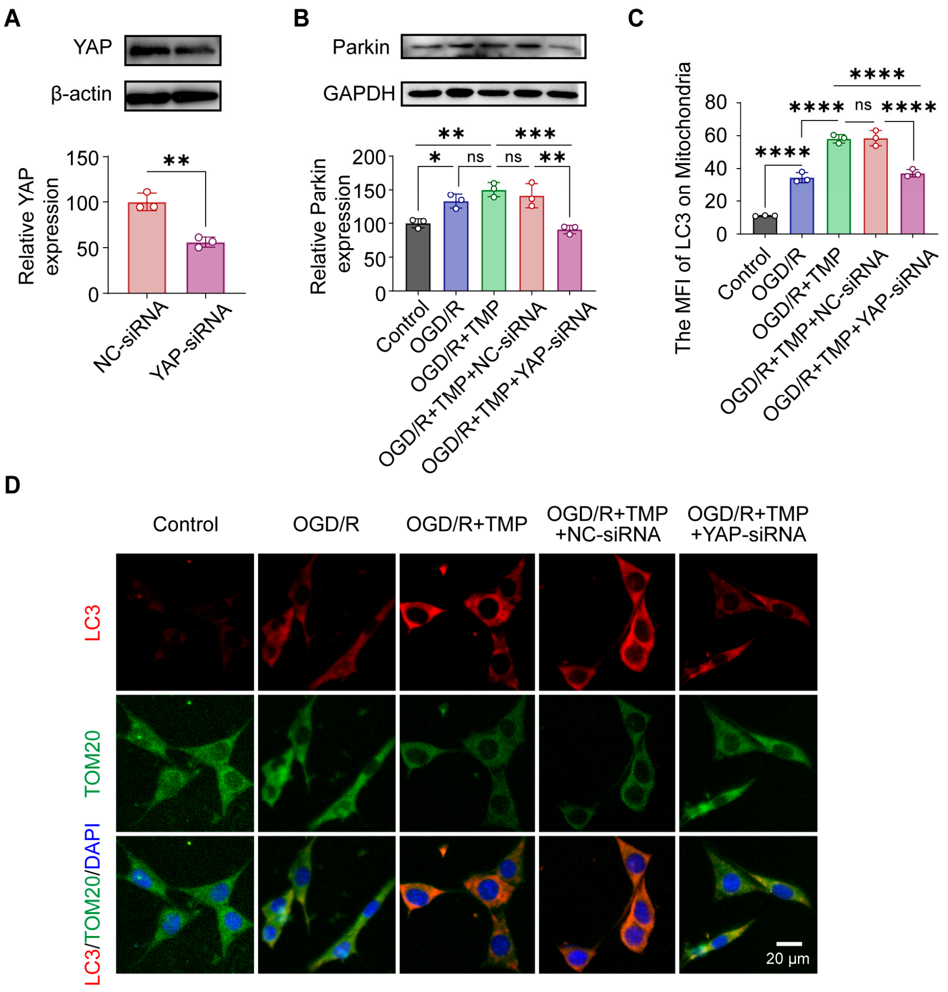

Supplementary Figure S6. YAP knockdown attenuates TMP-induced Parkin upregulation and mitophagy in HT22 cells after OGD/R.

(A) Western blot analysis confirming the knockdown efficiency of YAP siRNA in HT22 cells. Quantitative analysis of relative YAP expression normalized to β-actin. (B) Western blot analysis of Parkin expression in HT22 cells under the indicated treatments (Control, OGD/R, OGD/R + TMP, OGD/R + TMP + NC-siRNA, and OGD/R + TMP + YAP-siRNA). Quantification of Parkin expression normalized to GAPDH. (C) Quantification of the mitochondrial localization of LC3, expressed as the mean fluorescence intensity (MFI) of LC3 on mitochondria. (D) Representative immunofluorescence images showing LC3 (red), the mitochondrial marker TOM20 (green), and nuclei stained with DAPI (blue) in HT22 cells under the indicated conditions. Scale bar = 20 μm. n = 3 independent experiments. Data are presented as

mean ± SEM. $^{*}P < 0.05$, $^{**}P < 0.01$, $^{***}P < 0.001$, $^{****}P < 0.0001$; ns, not significant.
